# The Statin Paradox: Drivers and Consequences of Therapy Discontinuation

**DOI:** 10.3390/metabo16090603

**Published:** 2026-08-24

**Authors:** Adrianna Dylik, Mikołaj Musiał, Michał Radke, Dominika Tuzimek, Dominika Rogoża, Bartosz Czekała, Anna Wawrzyniak, Nadzeya Zhuk, Katarzyna Skrypnik, Damian Skrypnik

**Affiliations:** 1 Faculty of Medicine, Poznan University of Medical Sciences, 61-701 Poznań, Poland; adadylik@gmail.com; 2 Student Scientific Association of Lifestyle Medicine, The Student Scientific Society, Poznań University of Medical Sciences, 61-701 Poznań, Poland; musialmikolaj0@gmail.com (M.M.); formisradke@gmail.com (M.R.); dominikatuzimek@gmail.com (D.T.); dominika.rogoza38@gmail.com (D.R.);; 3 Day Psychiatric Rehabilitation Unit for Children and Adolescents, Institute of Psychoeducation Ltd., Bystra St. 7, Nowe Miasto, 61-366 Poznań, Poland; 4 Środa Hospital of the Sacred Heart of Jesus, 63-000 Środa Wielkopolska, Poland; 5 Polish Red Cross Maritime Hospital, Powstania Styczniowego Street 1, 81-519 Gdynia, Poland; 6 Multi-Specialty Independent Public Healthcare Complex in Nowa Sól, 7 Chałubińskiego Street, 67-100 Nowa Sól, Poland; 7 Department of Family Medicine, Poznan University of Medical Sciences, Przybyszewskiego Street 49, 60-355 Poznań, Polanddamian.skrypnik@gmail.com (D.S.); 8 Department of Human Nutrition and Dietetics, Poznań University of Life Sciences, Wojska Polskiego Street 28, 60-637 Poznań, Poland

**Keywords:** statins, statin discontinuation, medication adherence, polypharmacy

## Abstract

**Highlights:**

**What are the main findings?**

- Statin discontinuation is particularly frequent within the first year of therapy.
- Fear of side effects, misinformation, and poor doctor-patient communication are the primary drivers of statin non-adherence.

**What are the implications of the main findings?**

- Unaddressed non-adherence substantially increases long-term cardiovascular risks and overall healthcare costs.

**Abstract:**

Background: Statins are among the most frequently prescribed medications worldwide, with proven benefits in reducing all-cause and cardiovascular mortality, preventing major adverse cardiovascular events (MACEs), and lowering healthcare costs. Despite their well-established safety and effectiveness, discontinuation of statin therapy remains a common problem in both primary and secondary prevention. Methods: A systematic literature review was conducted in accordance with PRISMA guidelines and registered in the PROSPERO database (CRD420261295172). Original studies published since 1 January 2014 were identified through searches of PubMed (MEDLINE) and Google Scholar using predefined keywords. Methodological quality and risk of bias of the included observational studies were evaluated using the Newcastle-–Ottawa Scale (NOS) and Joanna Briggs Institute (JBI) Critical Appraisal Tools. Results: Synthesized data from 29 included studies indicate that up to half of patients discontinue statin therapy within the first year (discontinuation rates ranging from 27.1% to 47.0% in primary prevention and 18.5% to 32.4% in secondary prevention), with rates increasing over time. Major factors contributing to non-adherence include fear of side effects, particularly statin-associated muscle symptoms (SAMSs), misinformation, limited patient–physician communication, socioeconomic barriers, and polypharmacy. Discontinuation is associated with significantly higher all-cause and cardiovascular mortality (Hazard Ratios ranging from 1.30 to 4.65), higher rates of cardiovascular and cerebrovascular events, worsening metabolic outcomes, reduced quality of life, and greater healthcare expenditures. Conclusions: Improving adherence requires better patient education, addressing misconceptions, strengthening patient–physician relationships, and optimising treatment regimens. A multidisciplinary approach is needed to prevent unjustified discontinuation and improve long-term clinical outcomes.

## 1. Introduction

Statins are among the most frequently prescribed medications, indicated for both primary and secondary cardiovascular prevention due to their lipid-lowering and pleiotropic anti-inflammatory properties. Recent advances in lipid-lowering pharmacological strategies and novel therapeutic targets further underscore the importance of maintaining consistent lipid control to lower major adverse cardiovascular events [1,2]. Their use has significantly increased: in the United States, the number of prescriptions increased from 31 to 92 million between 2008 and 2018 [3], and globally, around 145.8 million people were taking statins in 2018 [4]. Statins reduce all-cause and cardiovascular mortality and lower the risk of cardiovascular events [5,6,7,8,9,10,11,12,13]. They improve lipid profiles by reducing low-density lipoprotein (LDL) cholesterol, improving overall metabolic control [5,10,12,14]. Beyond physical health, statins contribute to higher quality of life, better well-being, and lower long-term healthcare costs due to fewer hospitalizations and medical interventions [5,12,15].

Despite these benefits, statin discontinuation is common. A 2014 survey reported that 47% of primary prevention and 41% of secondary prevention users stopped statin therapy [16]. In a 2023 cohort, 6.1% of patients’ post-coronary revascularization stopped at 1 year and 21.4% after 7 years [17]. Attitudes among older adults are concerning: in an Australian study, 85% were willing to deprescribe at least one medication, and 95% preferred to stop statins [18]. Notably, one-third of patients did not inform their provider about discontinuation [19]. Discontinuation of statin therapy is associated with increased mortality and cardiovascular events. This clinical deterioration is mediated not only by the loss of lipid control but also by sudden vascular inflammation [20,21]. The reasons for discontinuation include perceived or real adverse effects—especially muscle and joint pain [17,18,19,22,23]—as well as doubts about necessity, worsening health, and comorbidities [17,24,25]. Although statins are among the safest and most studied drugs, concerns remain widespread.

While individual studies have evaluated reasons for poor adherence, a comprehensive synthesis integrating both patient-level behavioral drivers and downstream clinical and economic outcomes remains lacking. Therefore, this review aims to systematically analyze the determinants of statin discontinuation and quantify its clinical consequences across primary and secondary prevention cohorts.

To ensure clinical and conceptual clarity, this review distinguishes between key terminology: non-adherence (sub-optimal or irregular medication intake), discontinuation (complete cessation of statin therapy), and statin withdrawal syndrome (an acute rebound state marked by endothelial dysfunction and elevated vascular inflammation following abrupt statin cessation) [20,21].

## 2. Materials and Methods

### 2.1. Study Protocol and Eligibility Criteria

This systematic review was conducted in accordance with the Preferred Reporting Items for Systematic Reviews and Meta-Analyses (PRISMA) 2020 guidelines. The study protocol was prospectively registered in the PROSPERO database (Registration ID: CRD420261295172).

Studies were selected based on the following explicit Population, Exposure, Comparator, Outcome, and Study Design (PICOS) framework:

– Population: Adult patients (aged ≥ 18 years) prescribed statin therapy for either primary or secondary cardiovascular disease (CVD) prevention in real-world clinical settings.
– Exposure: Statin therapy discontinuation (defined as complete treatment cessation or a medication gap > 60–90 days), statin withdrawal, or non-adherence.
– Comparator: Continuous, persistent statin users (e.g., proportion of days covered [PDC] ≥ 80%).
– Outcomes: Primary outcomes included major adverse cardiovascular events (MACE), overall mortality, and cardiovascular-related mortality. Secondary outcomes encompassed patient-reported drivers of cessation (e.g., statin-associated muscle symptoms [SAMS], fear of adverse effects), healthcare resource utilization, and economic costs.
– Study Design and Limits: Original, peer-reviewed human observational studies (prospective/retrospective cohort, case–control, and cross-sectional studies) published in English between 1 January 2014 and the search date. Case reports, systematic/narrative reviews, conference abstracts, non-English publications, and studies with duplicated data were excluded.

### 2.2. Search Strategy and Selection Process

A comprehensive literature search was executed across the PubMed (MEDLINE), Google Scholar and Web of Science databases using combinations of Medical Subject Headings (MeSH) and free-text terms. The complete search string applied in PubMed was as follows: (“statins” AND (“Medication Adherence” OR “discontinuation” OR “non-adherence” OR “nonadherence” OR “deprescribing”) AND (“Cardiovascular Diseases” OR “Mortality” OR “cardiovascular events” OR “myocardial infarction” OR “stroke” OR “mortality”)). Full database-specific search strings and applied filters are detailed in Supplementary Table S1.

**Figure A1:**
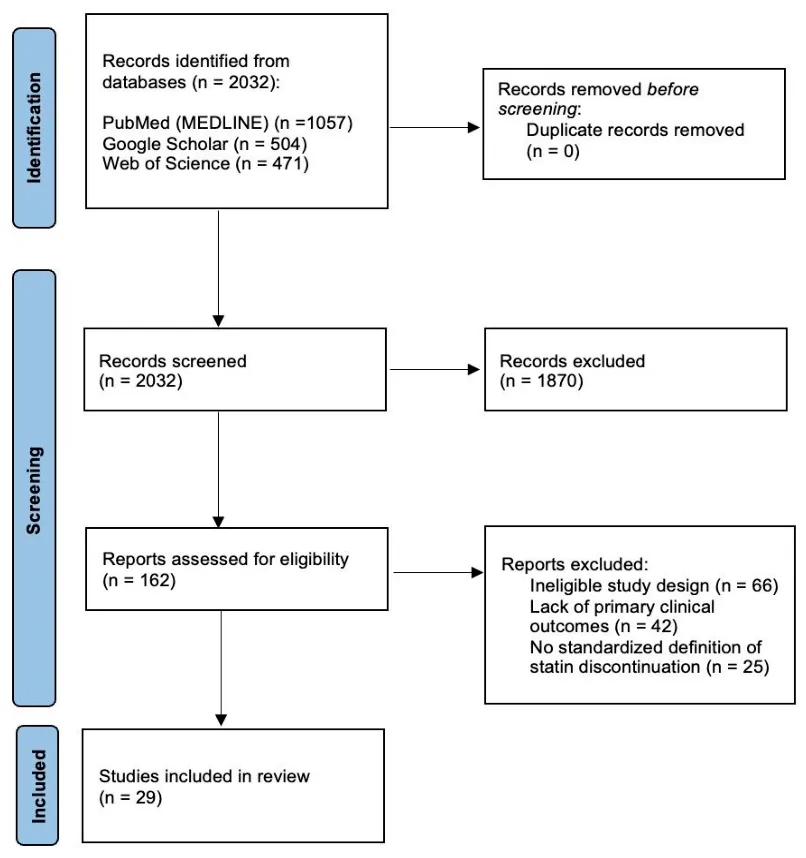
PRISMA flowchart.

The database searches returned a total of 2032 studies. After screening titles and abstracts, 1870 studies were excluded. Six independent researchers evaluated the full texts of the remaining 162 articles, resulting in the exclusion of 133 studies. A total of 29 original studies met all eligibility criteria and were included in this review. The step-by-step selection flow is illustrated in the PRISMA flowchart (Figure A1).

### 2.3. Quality and Risk of Bias Assessment

To evaluate methodological quality and potential risk of bias across included observational studies, we applied the Joanna Briggs Institute (JBI) Critical Appraisal Tools (for cohort [26], cross-sectional [27] and qualitative [checklist for qualitative studies [28]) and the Newcastle-Ottawa Scale (NOS) [29] tailored to the respective study designs. Studies were evaluated across key domains including sample representativeness, exposure measurement rigor, adjustment for confounding variables, outcome measurement reliability, and adequacy of follow-up. Based on NOS scores (0–9 scale), studies were categorized as low (7–9), moderate (4–6), or high (≤3) risk of bias.

### 2.4. Data Extraction and Outcome Measures

Data were systematically extracted, capturing study design, geographical location, sample size, population characteristics, definitions of discontinuation, identified drivers, and outcome metrics. The primary outcome measures were the drivers and clinical consequences of statin discontinuation, specifically major adverse cardiovascular events (MACE), all-cause mortality, cardiovascular hospitalizations, severe complications, quality of life changes, and healthcare costs. Statistical effect measures extracted primarily included Hazard Ratios (HR), Odds Ratios (OR), Relative Risks (RR), and correlation coefficients with their corresponding 95% Confidence Intervals (CI).

The study bias assessment analysis is presented in Table 1, Table 2 and Table 3.

## 3. Results

The baseline characteristics, study designs, sample sizes, identified drivers, and main clinical outcomes of all included studies (*N* = 29) are summarized in Table 4.

### 3.1. Determinants of Statin Discontinuation and Non-Adherence

The decision to discontinue or avoid statin therapy is influenced by a complex interplay of demographic, socioeconomic, clinical, and media-related factors.

#### 3.1.1. Demographic and Socioeconomic Determinants

Studies assessing the impact of age on statin adherence yield varying conclusions. Rea et al. [5] found that 4203 out of 29,047 patients using multiple drugs discontinued statins exclusively. The average age of these individuals was 76.5 years, indicating a higher tendency for older adults to discontinue statins. This finding is consistent with another study [16]. Patients aged <50 years and >75 years old demonstrated a greater likelihood of ending statin therapy. Contrary to these findings, Qi et al. [18] observed no significant correlation between age and the decision to discontinue statins. However, older patients were less inclined to stop taking statins, likely due to their heightened perception of cardiovascular mortality risk. In contrast, younger individuals were more likely to stop statin therapy because they perceived a lower risk of cardiovascular complications [18,22].

Regarding sex differences, in their analysis of the 4203 patients who discontinued statins, Rae et al. [5] noted that the majority were men (2507; 59.7%). Consistently, another study also reported that men were more prone to discontinue statin treatment [24]. However, some studies have presented opposing evidence, showing that women were more likely to stop statin use, while men were more willing to adhere to therapy [16,23,25]. Furthermore, Aivo et al. [12] noted that older women were less likely to receive early statin therapy following ischemic stroke. Conversely, Halava et al. [22] found no gender differences in statin discontinuation rates.

Socioeconomic status and financial parameters further modulate adherence. A low socioeconomic status often discourages individuals from seeking medical care, stemming from limited access to physicians or fear of judgment by medical professionals. In line with this view, a study reported a higher risk of statin discontinuation among individuals residing in large cities (>100,000 residents) compared with those in smaller towns or rural areas [24]. In contrast, Halava et al. [22] found no association between the socioeconomic status or educational level and statin discontinuation. However, patients with higher out-of-pocket costs or those prescribed higher doses of statins were more likely to discontinue therapy due to the financial burden. Booth et al. [30] found that low-income individuals were more likely to stop treatment.

#### 3.1.2. Clinical Health Status, Polypharmacy, and Patient Well-Being

A patient’s health condition and the number of medications they take often influence their ability to adhere to statin therapy. Rea et al. [5] reported that 4203 out of 29,047 individuals aged >65 years old who were using multiple medications (lipid-lowering, blood pressure-lowering, antidiabetic, or antiplatelet agents) discontinued statins during the observation period while continuing other treatments. Discontinuation was identified by a failure to fill prescriptions within 90 days of their expiration. The cohort of older patients experiencing polypharmacy often had comorbidities, including cerebrovascular disease (8.9%), ischemic heart disease (19.5%), heart failure (8.1%), respiratory disease (9.3%), kidney disease (5.7%), liver disease (0.5%), or cancer (5.1%) [5]. Similarly, Aivo et al. [12] revealed that early statin use following ischemic stroke was less common among patients with atrial fibrillation, heart failure, rheumatic disease, renal failure or mental disorders. Conversely, Halava et al. [22] found that vascular comorbidities reduced the likelihood of statin discontinuation, whereas antidepressant use and cancer history had no significant impact. Consistently, Lilly et al. [25] showed that patients with cardiovascular diagnoses and those on higher statin doses were more adherent. Additionally, Booth et al. [30] found that patients were more likely to stop statins if they had never used them before.

Patient well-being is a critical determinant of medication adherence. It can be speculated that discontinuation of statins was driven by severe side effects [5]. However, extensive research has shown that serious adverse effects of statins are rare. These include myopathy, rhabdomyolysis and severe liver damage, which occur in <1%, <0.1%, and around 0.001% of patients annually, respectively [5]. Halava et al. [22] reported similar findings. Nevertheless, there is no clear evidence explaining why patients would discontinue only statins while maintaining other medications, which may also have side effects [5]. Additionally, Qi et al. [18] found that frail older adults were less likely to stop statins compared with robust older adults.

Vinogradova et al. [16] reported that factors such as type 1 diabetes, smoking, and chronic liver disease were linked to an increased risk of statin discontinuation. Similarly, Vitturi et al. [11] noticed that severe symptoms also increased the risk of stopping statins.

#### 3.1.3. Media Influence and Communication Barriers

As demonstrated by Vitturi et al. [11], negative media reports about statins significantly discourage patients from using them. In addition to media, patient–physician interactions play a critical role in treatment adherence. According to Brinton et al. [36], only 45% of respondents discussed challenges related to statin use with their physicians. Moreover, just 25% reported receiving information about potential interactions between statins and other medications, while 39% admitted to not asking any questions and simply taking prescribed medications. This passive approach often leads patients to seek information independently. Brinton et al. [36] showed that 26% of patients using statins reported searching for information on statin therapy online, thereby increasing their exposure to inaccurate or incomplete data. Furthermore, Qi et al. [18] found that among 180 patients, 37% reported hearing negative information about statins, with 95% attributing it to the media.

While these findings provide valuable initial insights, several included studies exhibited a high risk of bias, suggesting that these specific observations should be interpreted with caution and require confirmation in methodologically robust research.

An overview of the collected data is shown in Table 5.

### 3.2. Patient Beliefs and Reasons for Non-Adherence

Six studies used different methods to identify the most important reasons for both primary and secondary non-adherence to statin therapy. All studies showed that an important factor pushing patients towards statin discontinuation or primary non-adherence is worry about side effects, mostly related to muscles and joints.

In a survey performed by Wouters et al. [23], patients were motivated to discontinue treatment mostly due to a lack of knowledge and conviction that statins are effective and necessary. The patients also stressed that practical problems such as difficulties or lack of proper instructions about taking pills, low income, and poor communication may create an obstacle in successful statin treatment. Each practical problem and concern towards a side effect increased the risk of statin non-adherence.

According to Tjia et al. [37], patients with terminal diseases described potential benefits from statin discontinuation such as saving money, stopping other medications, improving their quality of life, and reducing the number of symptoms they are already experiencing. Patients with a primary diagnosis of cardiovascular disease noted the potential benefits of statin discontinuation more frequently.

As demonstrated in the STAY study, media coverage exaggerating non-life-threatening side effects, combined with a fundamental lack of patient awareness regarding hypercholesterolemia risks, represents a primary catalyst for premature statin discontinuation [40].

Physician-reported data further underline the role of adverse effects as the primary driver of treatment interruption. In a qualitative study evaluating primary care physician (PCP) perspectives, side effects—predominantly muscle- and joint-related symptoms—were identified as the single most prominent reason given by patients for statin discontinuation (accounting for 47% of total weighted votes). Although statin re-challenge was the most common clinician response following treatment cessation (31% of votes), PCPs consistently cited patients’ apprehension and fear of adverse effects as the main barrier to successful therapy re-initiation (70% of votes) [42].

Older adults who took statins to prevent primary cardiovascular disease claimed that among the main reasons for their statin discontinuation would be willingness to reduce the number of medications they take [38]. A self-estimated low cardiovascular risk was also a risk factor for non-adherence to statin therapy. Patients who declared they did not feel any positive or negative statin effects would be more willing to choose to discontinue statins.

Finally, Tarn et al. [19] interviewed patients who reported primary non-adherence. The participants expressed their wish to improve their lipid profile by alternative ways such as lifestyle changes or dietary supplements before being put on medications. Similarly to patients examined by Brunner et al. [38], the participants interviewed by Tarn et al. [19] estimated that they were too young or in too good of health to take statins. They also noted that being seen as part of medical guidelines rather than as individuals led to mistrust in their physicians’ recommendations and barriers in communication. Moreover, they had bad previous experiences with medications and believed that high cholesterol levels are genetic and therefore do not require treatment. However these results should be interpreted cautiously due to methodological limitations.

**Figure A2:**
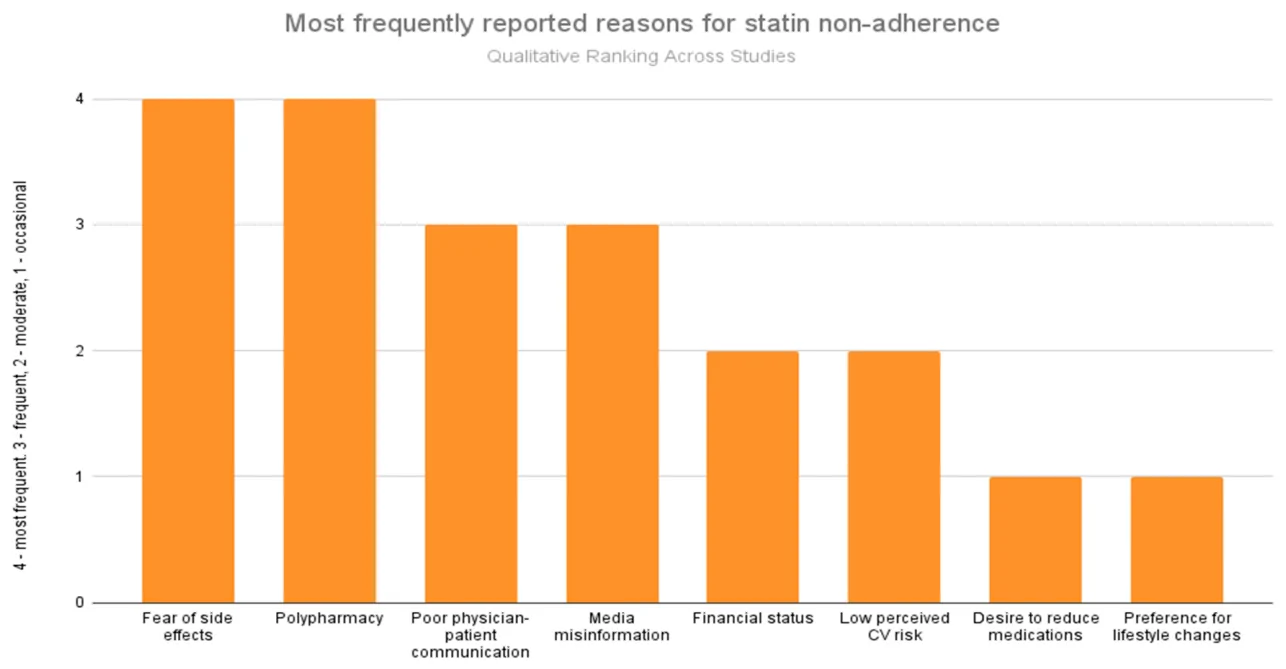
Most frequently reported reasons for statin non-adherence.

An overview of the collected data is shown in Table 6. Most frequently reported reasons for statin non-adherence are presented in Figure A2.

### 3.3. Clinical and Economic Consequences of Statin Discontinuation

#### 3.3.1. Mortality and Cardiovascular Events

Multiple studies have shown that statin users experience lower mortality rates compared with non-statin users and those who discontinue statin therapy [9,10,12,13]. Statin discontinuation is connected to an increased mortality rate (HR ranging from 1.15 to 4.65), sometimes resulting in the same or higher risk as if the patients had never used statins. This is particularly crucial after events such as ischemic stroke, percutaneous coronary intervention (PCI), or acute myocardial infarction (AMI), as discontinuation after such incidents can lead to complications, a higher risk of death, or health problems [9,10,11,13]. Even short-term statin discontinuation has been associated with a higher rate of all-cause mortality (HR = 1.83; 95% CI: 1.58–2.12) [12]. One study showed that statin discontinuation did not increase mortality, but it considered a relatively small sample size [8].

Thompson et al. [6] mentioned that patients who discontinued long-term statin treatment had a higher risk of major adverse cardiovascular events (HR = 1.33; 95% CI: 1.19–1.49) than those who continued treatment. Giral et al. [7] showed that patients aged ≥75 years, without prior cardiovascular disease and who discontinued using statins, reported a 33% higher risk of hospitalizations for cardiovascular events over 2.5 years than those who consistently took statins (HR = 1.33; 95% CI: 1.24–1.41). Tong et al. [10] reported that patients who stopped statins within three weeks after intravenous thrombolysis were less likely to achieve favorable long-term outcomes (OR = 2.68; 95% CI: 1.31–5.47). Additionally, ceasing statin therapy after myocardial infarction or PCI correlated with worse clinical outcomes and an increased risk of adverse events (HR = 1.48; 95% CI: 1.21–1.81) [30].

A phenomenon called ‘statin withdrawal syndrome’ suggests that abrupt discontinuation of high-intensity statins may result in a rebound effect that exacerbates cardiovascular risk. Gradual dose reduction may diminish these effects and has less risk compared with abrupt discontinuation [30]. According to the guidelines presented by Kim et al. [13], poor adherence to statin therapy consistently results in worse clinical outcomes compared with continued use (1-year MACE HR = 1.71; 95% CI: 1.32–2.22).

#### 3.3.2. Cerebrovascular, and Metabolic Outcomes

Statin discontinuation increases the risk of adverse cerebrovascular events. Patients who discontinue therapy have a higher risk of ischemic stroke and death compared with those who continue treatment [6].

In patients with acute ischemic stroke, good adherence to statins is associated with better functional outcomes and lower recurrence rates, whereas discontinuation increases the risk of stroke recurrence (OR = 2.41; 95% CI: 1.35–4.31) [6]. These findings emphasize the importance of continuous statin use for improving recovery and reducing recurrence.

Early initiation of statins after embolic stroke significantly reduces recurrence risk and improves outcomes [11]. Similarly, the absence of early statin therapy after ischemic stroke is associated with increased risk of recurrent events, regardless of patient characteristics or other preventive measures [12]. Importantly, continued statin use does not increase the risk of intracerebral hemorrhage, supporting its safety in stroke prevention.

Statins positively influence metabolic health by improving lipid profiles and reducing LDL cholesterol levels via inhibition of 3-hydroxy-3-methylglutaryl coenzyme A reductase (HMG-CoA) [10]. This benefit extends to overall metabolic parameters, contributing to better lipid management and metabolic control in statin users [5,10,12]. Additionally, statins have beneficial pleiotropic effects on the endothelium, immune system, platelets, and vascular smooth muscles, and they have been shown to stabilize atherosclerotic plaque [8,12]. In contrast, non-statin users and those who discontinue statin therapy exhibit poorer lipid profiles and metabolic health. Discontinuation may contribute to a deterioration in lipid metabolism, resulting in worsening metabolic parameters in the long term, such as a higher Killip class (≥III) and increased levels of peak troponin-I, N-terminal pro-brain-type natriuretic peptide, and high-sensitivity C-reactive protein [13].

#### 3.3.3. Economic Outcomes

Patients on statin therapy report higher quality of life compared with non-users and those who discontinue treatment [11]. Beyond physical health, statins positively affect well-being and life satisfaction. Although some patients may experience side effects such as muscle pain (<0.1% annually) or fatigue, discontinuation is associated with a decline in quality of life due to increased risk of cardiovascular events, stroke, and myocardial infarction [5,13].

In patients after ischemic stroke or receiving thrombolysis, statin discontinuation is linked to poorer long-term outcomes compared with continued therapy, resulting in reduced quality of life and more frequent hospitalizations [12,13].

Statin users experience lower overall medical costs in the long term due to the preventive benefits of statin therapy, which can reduce the risk of cardiovascular incidents, strokes, and neurological incidents. This may be associated with fewer hospitalizations and less intensive medical interventions. However, patients who discontinue statins tend to have increased medical costs. In statin users who discontinue statin therapy, the initial reduction in cardiovascular risk eventually disappears, which contributes to a higher number of cardiovascular events and associated healthcare expenses [5].

An overview of the collected data is shown in Table 7.

### 3.4. The Role of the Physician and Healthcare System in Statin Adherence

Physicians and the medical system play a significant role in ensuring patients adhere to taking their medications, including long-term statin therapy.

An important factor in statin adherence is the identification of patients who have statin-associated symptoms (SAS), followed by modification of their treatment. In a cohort study carried out in 12 countries with the help of 810 clinicians, 72% of the patients presented symptoms similar to SAS; however, after verification, only 6% were diagnosed with SAS. Of these patients, 52% received a lower dose of statin with or without other lipid-lowering treatment, and 38% were prescribed with another lipid-lowering treatment. Thus, all patients with SAS maintained primary or secondary prevention [39]. These determinants were primarily derived from studies with higher potential for selection and reporting bias, highlighting the need for further high-quality, standardized studies.

The frequency of general practitioner visits also has great importance. In a study from Western Australia, patients who visited their general practitioner at least three times per year had a 47% lower chance of statin discontinuation compared with patients who only attended only once. This effect occurred in both new and long-term statin users [31].

Collaboration between cardiologists, primary care physicians, and nurses improves adherence. In patients after acute myocardial infarction, combined follow-up within 30 days resulted in higher adherence compared with single-provider care [32]. This highlights the importance of coordinated care, consistent communication, and shared treatment plans in improving outcomes.

Face-to-face education by pharmacists can also form part of a multidisciplinary approach to decrease statin discontinuation. In this approach, pharmacists can help patients understand why they need to take their medications. In a study conducted in West Virginia, including patients aged 40–75 years who had not had prescribed statin within the previous year, 80% of the patients were able to enumerate at least one benefit of statin intake after receiving education from a pharmacist. Before the study, 30% of the patients were unsure of whether they should take statins prescribed to them; this percentage increased to 80% after the study [41].

Effective patient–physician communication remains essential. Many patients report insufficient information about statin therapy, including its purpose, mechanism, and potential interactions [36]. Establishing a therapeutic relationship and providing clear, evidence-based education improves adherence and reduces discontinuation risk [33].

From the primary care perspective, re-initiating statin therapy following discontinuation remains clinically challenging. Primary care physicians identify patients’ fear of adverse events—predominantly subjective muscle and joint discomfort—as the single greatest obstacle to treatment re-challenge, highlighting the profound operational impact of the nocebo effect in routine practice [35].

The duration and frequency of medical consultations are crucial to the effectiveness of therapy. The initial visit should include a precise definition of the therapeutic goals, a discussion of the benefits and potential risks of the therapy, and an opportunity for the patient to express any concerns about the treatment. Subsequent visits should be used for monitoring and education: verifying that the established goals have been achieved, supplementing the patient’s knowledge, and early identification of adherence issues. Regular meetings foster trust, increase patient motivation to continue treatment, and enable optimization of therapy over time. This is particularly important in the elderly population, for whom changes in pharmacotherapy and lifestyle may require gradual support [31,36].

Trust in one’s physician is also a significant factor regarding whether patients continue statin therapy. A survey conducted on patients aged ≥65 years showed that 94% of them trusted their general practitioner and were willing to continue the statin treatment if their general practitioner told them they needed to do so. Only 25% of them wanted to discuss it with friends or family [38]. Positive patient motivation from treating physicians also helps improve adherence to therapy. Instead of focusing on fear-based arguments such as potential complications, it is more effective to emphasize the benefits of taking medication on a regular schedule and following a healthy lifestyle. Systematically reassuring patients that taking statins after a myocardial infarction can improve their quality of life, reduce their risk of recurrence, and allow for longer-term functional recovery promotes a sense of empowerment and engagement in treatment. An approach based on support and education contributes to greater patient compliance and reduces the risk of treatment discontinuation. Discussing the patients’ concerns about treatment is also crucial, so patients feel fully prepared when starting treatment. Research shows that patients respond better to positive messages, building trust and a sense of control over their health, than to threats and warnings [36,38]. The simplicity of drug use is another crucial factor. Indeed, single-pill solutions have been implemented to increase compliance to statin therapy. A retrospective analysis conducted on 1219 patients prescribed statins showed that the number of medications the patient has to take plays a crucial role in statin adherence. A single-pill combination of ezetimibe and rosuvastatin had higher patient compliance (75.2%) compared with having to take each pill separately (51.8%) [34].

An overview of the collected data is shown in Table 8.

## 4. Discussion

To evaluate the literature accurately, a fundamental distinction must be drawn between unguided non-adherence and clinically rational deprescribing. Statin cessation encompasses a broad spectrum of clinical scenarios ranging from premature, patient-initiated drop-out to structured, physician-led medication withdrawal. Inappropriate or unjustified non-adherence refers to treatment cessation by patients who maintain a clear cardiovascular risk-reduction benefit, typically mediated by misconceptions regarding safety, media-driven anxiety, or low perceived necessity. Conversely, clinically justified deprescribing represents a planned, patient-centered process of medication rationalization. In contexts such as terminal illness, severe frailty, high polypharmacy burden, or where the time-to-benefit of statin therapy exceeds overall life expectancy, deprescribing is a medically sound strategy aimed at optimizing quality of life and reducing medication burden.

The clinical impact of statin discontinuation is highly heterogeneous and dictated by baseline absolute cardiovascular risk, clinical acuity, and treatment indication. In secondary prevention settings—particularly among post-acute myocardial infarction (AMI), post-percutaneous coronary intervention (PCI), and acute ischemic stroke cohorts—statin withdrawal represents an acute clinical hazard. In these high-risk populations, cessation disrupts active plaque stabilization, leading to a pronounced, early excess in recurrent ischemic events and short-term mortality.

In contrast, within primary prevention cohorts, the absolute risk reduction conferred by statins is lower, and the clinical sequelae of discontinuation manifest as a slow, cumulative loss of long-term cardiovascular protection rather than an immediate acute crisis. In older adults (≥75 or ≥85 years), risk stratification requires careful differentiation between secondary prevention (where net clinical benefit remains robust) and primary prevention in frail, multimorbid patients with limited life expectancy, where metabolic goal relaxation and structured deprescribing represent clinically appropriate care.

The current evidence demonstrates that continuing statin therapy, particularly among older individuals and patients with high baseline cardiovascular risk, significantly lowers the incidence of major adverse cardiovascular events (MACE) and overall mortality. Discontinuation is particularly crucial in acute medical conditions and is a growing problem among patients. In the first six months, as many as 25–50% of patients discontinue treatment, which may be associated with an increased risk of cardiovascular disease, atherosclerosis, myocardial infarction, or stroke, especially in people with risk factors. Studies are inconsistent regarding the gender and average age of individuals who discontinue therapy. Older patients may be less likely to stop taking statins, possibly due to their heightened perception of cardiovascular mortality risk. Statin discontinuation may be more common in people with a low socioeconomic status who discontinue therapy for financial reasons.

Although patients may discontinue statins due to their cost, this can paradoxically lead to significantly higher long-term treatment costs, resulting from hospitalizations from recurrent myocardial infarction or other cardiovascular complications. Research indicates that discontinuing secondary therapy increases the risk of cardiac events, which generates additional financial burdens for both the healthcare system and the patient. Therefore, investing in patient education, financial support mechanisms, and adherence monitoring is not only clinically beneficial but also economically beneficial, minimizing the risk of complications and the associated treatment costs.

Additionally, poor patient–physician cooperation and the negative influence of the media and the Internet spreading contradictory medical information contribute to non-adherence to treatment. Another negative factor is polypharmacy, which is mainly caused by multimorbidity, especially prevalent in aging societies, and wide access to effective medications. It contributes to the use of many drugs at the same time [43]. The profile of a patient avoiding statins also includes chronic smokers and patients with type 1 diabetes.

There are several reasons why patients decide to discontinue statin therapy. The most reported is the fear of side effects caused by these medications, such as muscle pain and fatigue. In addition, the risk of liver dysfunction and kidney failure is increased, which can make patients afraid of taking statins [44]. The harmful influence of disinformation that can be found in mass media and the nocebo effect must also be mentioned, where negative expectations directly trigger physical symptoms despite the absence of true pharmacological toxicity.

Many causes of statin non-adherence are related to the patients’ personal beliefs and are inconsistent with scientific knowledge. Among them are a belief that statins are not efficacious or necessary, an incorrect self-estimated health status, and claiming that a condition is due to a genetic effect that cannot be treated with medication. One of the major underlying causes of these convictions seems to be that the positive statin effects are not actually noticeable by the user, just like hypercholesterolemia itself is usually asymptomatic [45,46].

However, it should be noted that much of the evidence describing patient-reported beliefs, media influence, and fear of side effects relies on cross-sectional surveys and qualitative designs (e.g., Qi et al., Brinton et al., Hovingh et al., Tarn et al., Clark et al.) which exhibit a high risk of selection, response, and reporting bias. While these observational data effectively capture subjective patient sentiment and clinical barriers, they cannot establish direct causality regarding whether these perceived concerns independently drive hard clinical discontinuation outcomes without residual confounding.

Therefore, information provided to patients should be based on solid scientific evidence to counteract common misunderstandings and misconceptions about statin therapy. The message should be clear, convincing, and tailored to the patient’s level of knowledge and expectations, considering their concerns and explaining the benefits of long-term therapy. This approach promotes better adherence and reduces the risk of cardiovascular complications [45].

Finally, there are practical problems associated with taking statins, including limited income, trouble taking pills, and communication problems. Moreover, statin therapy is long term. After years of taking these medications, patients may find the therapy inconvenient or not worth their effort.

After stopping statin treatment, LDL cholesterol levels rise, promoting the formation of atherosclerotic plaques, which can lead to vessel blockage and result in stroke or myocardial infarction [47]. This evidence strongly supports the continuation of statin therapy to reduce cerebrovascular risk and to improve functional outcomes after stroke. Statins also help stabilize these plaques, reducing the risk of rupture and the formation of emboli [48]. Discontinuing statins weakens these plaques, increasing the risk of rupture and complications. Additionally, statins positively affect other metabolic parameters, such as lowering triglycerides and raising high-density lipoprotein (HDL) cholesterol [48]. Stopping their use may lead to the development of metabolic disorders like insulin resistance or type 2 diabetes, which are risk factors for cardiovascular diseases [49,50]. Furthermore, statins have an anticoagulant effect, and their discontinuation increases the risk of blood clots and cerebrovascular events [47]. Therefore, discontinuing statin therapy can significantly worsen the patient’s health, leading to more frequent hospitalizations and higher mortality [51].

A notable limitation of this synthesis is that a substantial portion of the included literature—specifically cross-sectional surveys (e.g., Qi et al., Brinton et al., Hovingh et al.) and qualitative studies (e.g., Tarn et al., Clark et al.)—exhibited a high overall risk of bias. Consequently, while these studies offer valuable thematic insights into patient-reported reasons for non-adherence, their findings should be interpreted with caution as hypothesis-generating. In contrast, the adverse cardiovascular outcomes of statin cessation are more firmly established in prospective and cohort studies with lower risk of bias.

**Figure A3:**
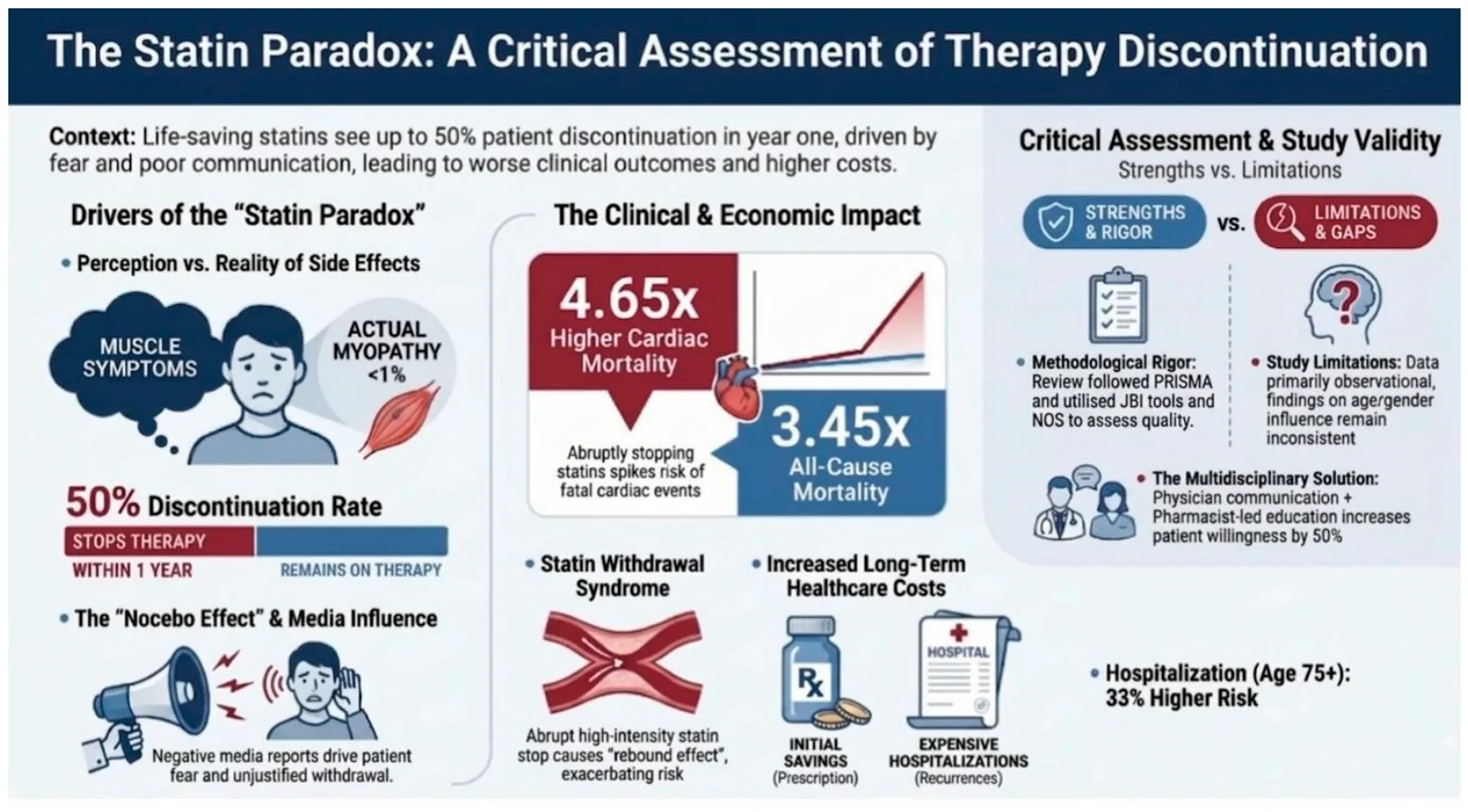
The critical assessment of statin therapy discontinuation and study bias.

The critical assessment of statin therapy discontinuation and study bias is presented in Figure A3.

## 5. Clinical Implications

It is essential to ensure patients continue statin therapy to reduce cardiovascular morbidity and mortality. Strategies to improve adherence should combine patient education, effective patient–physician communication, and systemic support [52]. Providing clear information about the proven benefits and safety of statins, as well as addressing misconceptions and the nocebo effect, is crucial [33,39]. Educational initiatives and public health campaigns may help counteract misinformation and reinforce trust in therapy.

At the clinical level, shared decision-making, prompt management of side effects, and regular monitoring can reassure patients and strengthen commitment to long-term treatment [42]. When patients present with muscle symptoms (SAMS), clinicians should utilize structured re-challenge protocols before permanently discontinuing therapy. Temporarily halting the statin and then restarting at a lower dose, switching to alternate-day dosing regimens (e.g., rosuvastatin), or adding non-statin lipid-lowering agents like ezetimibe allows up to 90% of reporting patients to successfully continue lipid-lowering therapy. Simplifying therapy by reducing polypharmacy, particularly through the use of single-pill fixed-dose combinations (FDCs), adjusting doses, or switching to better-tolerated statins may also prevent discontinuation. Systemic measures such as reimbursement programs, wider access to generics, and the involvement of pharmacists or nurses in follow-up care can further support adherence. Digital tools, including reminder systems, may provide additional assistance [52,53]. Overall, a multifaceted approach that integrates medical, psychological, and organizational strategies is required to minimize premature statin discontinuation and to improve long-term cardiovascular outcomes.

Importantly, many countries have implemented e-health systems and the ability to issue e-prescriptions, which provide therapeutic benefits such as reviewing the prescription history, monitoring medication dispensing, and easier access to medications. Physicians and pharmacists can partially track patient adherence and see whether a prescription has been filled. However, the ability to monitor prescription dispensing using electronic systems should not replace regular open communication between patients and physicians. During appointments, physicians have the opportunity to discuss the benefits and risks of therapy with the patient and to clarify any concerns. Such a conversation builds trust and tailors treatment. Although electronic systems increase transparency and convenience, they do not replace patient–physician communication. Therefore, electronic platforms and the ability to issue e-prescriptions should be viewed as a valuable tool supporting the treatment process, and its combination with a physician’s approach increases the chance of long-term therapeutic success.

## 6. Future Studies

Although much is known about the determinants of statin discontinuation, several areas require further investigation. Future research should clarify which patient-related factors—such as psychological traits, health literacy, or cultural influences—most strongly predict non-adherence, as current evidence on the roles of age, gender, and socioeconomic status remains inconsistent. A better understanding of the nocebo effect and patients’ perception of adverse effects is particularly important, as true statin intolerance is relatively uncommon. Studies distinguishing pharmacological intolerance from psychologically driven symptoms, and testing strategies to mitigate the nocebo effect, are needed. Moreover, clinical trials assessing the effectiveness of educational tools, digital reminders, and shared decision-making models could identify the most promising approaches to improving adherence. At the system level, evaluations of reimbursement policies, pharmacist- or nurse-led programs, and real-world data from electronic health records may provide valuable insights into long-term treatment patterns. In summary, future studies should integrate clinical, psychological, and organizational perspectives to develop effective, evidence-based strategies that prevent premature statin discontinuation and ultimately improve cardiovascular outcomes.

## 7. Strengths and Limitations

This review offers a broad and integrated overview of the factors influencing statin discontinuation, covering demographic, socioeconomic, clinical, and psychosocial determinants. A key strength is the multidimensional approach, which combines patient-reported reasons for non-adherence with evidence on clinical outcomes such as mortality, cardiovascular events, and quality of life. By also addressing pleiotropic effects and cost-effectiveness, this review highlights both the medical and economic consequences of statin discontinuation, providing valuable information for clinicians and policymakers.

However, several limitations should be acknowledged. The evidence base is heterogeneous, with inconsistent findings regarding age, sex, and socioeconomic influences, which reduces comparability and generalizability. Significant regional and geographic differences across healthcare systems further limit the direct application of findings across different populations. Many of the cited studies are observational, limiting causal interpretation, and several included cohorts relied on relatively short follow-up periods, which may underestimate long-term cardiovascular outcomes and sustained adherence patterns. Additionally, reliance on self-reported survey data in several primary studies introduces potential self-report bias, where patients may over- or underestimate their compliance or attribute symptoms to statins unrealistically. Furthermore, few studies evaluated targeted interventions aimed at improving adherence. Despite these constraints, the strength of this work lies in synthesizing diverse perspectives and identifying critical gaps, which sets the stage for future research and clinical strategies.

## 8. Conclusions

Statin discontinuation may be associated with an increased risk of cardiovascular and cerebrovascular events, potentially contributing to higher hospitalization rates and mortality. The factors influencing non-adherence appear to be multifactorial, encompassing potential demographic and socioeconomic influences, comorbidities, and polypharmacy, as well as perceived side effects, misinformation, and gaps in patient–physician communication. Consequently, attempts to address statin discontinuation could benefit from a multifaceted approach that integrates targeted patient education, shared decision-making, therapy optimization, and systemic support. Future research would be valuable to further explore and evaluate interventions aimed at sustaining long-term adherence.

## Figures and Tables

**Table 1:** The study bias assessment analysis (JBI Critical Appraisal Tools for cohort study and NOS).

Study Reference	Study Design	Selection Bias	Detection Bias	Confounding/Reporting Bias	Overall Risk of Bias
Rea et al. [5]	Retrospective Cohort	Low	Low	Moderate	Low/Moderate
Thompson et al. [6]	Retrospective Cohort	Low	Low	Moderate	Low/Moderate
Giral et al. [7]	Population Cohort	Low	Low	Moderate	Low/Moderate
Vitturi et al. [8]	Clinical Cohort	Moderate	Moderate	Moderate	Moderate
Jeong et al. [9]	Population Cohort	Low	Low	Moderate	Low/Moderate
Tong et al. [10]	Observational	Moderate	Moderate	Moderate	Moderate
Aivo et al. [12]	Retrospective Cohort	Low	Low	Moderate	Low/Moderate
Kim et al. [13]	Clinical Cohort	Moderate	Low	Moderate	Moderate
Vinogradova et al. [16]	Prospective Cohort	Low	Low	Low	Low
Yamamoto et al. [17]	Clinical Cohort	Moderate	Low	Moderate	Moderate
Halava et al. [22]	Cohort Study	Low	Low	Moderate	Low/Moderate
Nielsen et al. [24]	Prospective Cohort	Low	Low	Low	Low
Lilly et al. [25]	Cohort Study	Moderate	Low	Moderate	Moderate
Booth et al. [30]	Retrospective Cohort	Low	Low	Moderate	Low/Moderate
Seaman et al. [31]	Retrospective Cohort	Low	Low	Moderate	Low/Moderate
Hickson et al. [32]	Clinical Cohort	Moderate	Low	Moderate	Moderate
Sessa et al. [33]	Database Analysis	Moderate	Low	Moderate	Moderate
Perez de Isla et al. [34]	Retrospective Analysis	Low	Low	Moderate	Low/Moderate
Engell et al. [35]	Retrospective Cohort	Low	Low	Moderate	Low/Moderate

**Table 2:** The study bias assessment analysis JBI Critical Appraisal Tools for cross-sectional study.

Study Reference	Study Design	Selection Bias	Detection Bias	Confounding/Reporting Bias	Overall Risk of Bias
Qi et al. [18]	Cross-sectional	High	Moderate	High	High
Wouters et al. [23]	Survey/Refill data	Moderate	Moderate	Moderate	Moderate
Brinton et al. [36]	Online Survey	High	Moderate	High	High
Tjia et al. [37]	Cross-sectional	Moderate	Moderate	Moderate	Moderate
Brunner et al. [38]	Mixed-methods	High	Moderate	Moderate	Moderate/High
Hovingh et al. [39]	Clinician Survey	High	Moderate	High	High
Tokgözoğlu et al. [40]	Cross-sectional	Moderate	Moderate	Moderate	Moderate

**Table 3:** The study bias assessment analysis (JBI Critical Appraisal Tools for qualitative study).

Study Reference	Study Design	Selection Bias	Detection Bias	Confounding/Reporting Bias	Overall Risk of Bias
Tarn et al. [19]	Qualitative (FGs)	High	High	High	High
Clark et al. [41]	Educational Study	High	Moderate	High	High
Tanner et al. [42]	Qualitative (NGT)	Moderate	Moderate	Moderate	Moderate

**Table 4:** Main characteristics, determinants, and clinical outcomes of the included studies (*N* = 29).

First Author and Year	Study Design and Country	Sample Size (*N*) and Population	Discontinuation/Non-Adherence Definition	Identified Drivers/Determinants	Main Clinical and Economic Outcomes (Effect Size, 95% Cl)	Quality Score and Assessment Tool
Rea et al. (2021) [5]	Retrospective nested case–control (Italy)	*N* = 29,047 (Older adults ≥ 65 yrs, Primary and Secondary, Polypharmacy)	Complete statin discontinuation (no refill for ≥60 days)	Polypharmacy burden, advanced age, asymptomatic clinical status	Increased risk of heart failure hospitalization (HR = 1.24; 95% CI: 1.07–1.43), MACE (HR = 1.14; 95% CI: 1.03–1.26), and overall mortality (HR = 1.15; 95% CI: 1.02–1.30)	NOS: 8/9
Åivo et al. (2023) [12]	Population-based retrospective cohort (Finland)	*N* = 7249 (Secondary prevention—Ischemic stroke)	Lack of statin therapy post-discharge or pre-stroke discontinuation	Inadequate post-stroke discharge management, low treatment initiation	Significantly higher 1-year all-cause mortality (HR = 1.83; 95% Cl: 1.58–2.12) and recurrent ischemic stroke	NOS: 8/9
Halava et al. (2016) [22]	Prospective cohort study (Finland)	*N* = 12,548 (Primary prevention)	Discontinuation within the first year of initiation (no refill ≥ 365 days)	Low baseline cardiovascular risk, low income, living alone, absence of hypertension/diabetes co-therapies	Early drop-out form preventive therapy leading to long-term cumulative loss of cardiovascular prevention	NOS: 7/9
Tjia et al. (2017) [37]	Multicenter prospective cohort/trial subgroup (USA)	*N* = 381 (Patients with life-limiting illness/Palliative setting)	Physician-guided or patient-initiated statin deprescribing/cessation	Advanced illness, focus on comfort care, reduction in pill burden, physician recommendation	No increase in short-term cardiovascular mortality; improved quality of life and reduced overall medication costs	NOS: 8/9
Wouters et al. (2016) [23]	Cross-sectional survey and pharmacy data (Netherlands)	*N* = 970 (Primary and Secondary prevention)	Non-adherence defined as PDC <80% or self-reported intentional non-adherence	Negative beliefs about statins, fear of adverse events, low necessity beliefs, lack of shared decision-making	High rates of intentional cessation; necessity-concerns framework strongly predicted adherence behavior	JBI: 8/9
Brunner et al. (2024) [38]	Mixed-methods study (Survey and Interviews) (Switzerland)	*N* = 312 (Older adults ≥ 70 yrs, Primary prevention)	Statin discontinuation or reluctance to continue therapy	Perception of low personal benefit, advanced age, absence of symptoms, doctor’s recommendation to stop	High willingness to deprescribe among older adults when initiated by a physician; low self-perceived risk of CVD events	JBI: 9/10
Vinogradova et al. (2016) [16]	Prospective open cohort study (QResearch) (UK)	*N* = 45,793 (Primary and Secondary prevention)	Statin discontinuation (gap > 90 days without prescription refill)	Media coverage, muscle symptoms, lower baseline CVD risk, polypharmacy	Discontinuation associated with increased risk of overall mortality (HR = 1.42; 95% Cl: 1.34–1.50) and cardiovascular events	NOS: 9/9
Nielsen and Nordestgaard (2016) [24]	Nationwide prospective cohort (Denmark)	*N* = 671,000 (Primary and Secondary prevention)	Statin cessation within 6 months of initiating therapy	Exposure to negative statin-related news stories in national media	Increased risk of myocardial infarction (OR = 1.26; 95% Cl: 1.21–1.31) and cardiovascular death (OR = 1.18; 95% Cl: 1.14–1.23)	NOS: 9/9
Brinton (2018) [36]	Patient survey (ACTION study) (USA)	*N* = 1014 (Statin users &Primary and Secondary & Secondary)	Discontinuation due to patient choice or side effects	Statin-associated muscle symptoms (SAMS), poor physician communication, fear of organ damage	Over 50% of patients who stopped statins did so without consulting their physician; highlights critical communication gap	JBI: 7/0
Qi et al. (2015) [18]	Cross-sectional survey (Australia)	*N* = 383 (Older adults ≥ 65 yrs, Primary and Secondary)	Willingness to have statins deprescribed/non-adherence	High pill burden (polypharmacy), perception of statin necessity, trust in physician	88.3% of participants were willing to stop statins if recommended by their physician; highlights the critical role of physician-led deprescribing	JBI: 8/9
Tarn et al. (2021) [19]	Mixed-methods study (Interviews and Surveys) (USA)	*N* = 189 (Primary non-adherence, Primary and Secondary)	Primary non-adherence (failing to fill initial statin prescription within 60 days)	Fear of adverse effects, lack of perceived need, cost concerns, poor patient-provider communication	Primary non-adherence is strongly driven by unaddressed patient skepticism and lack of clear explanation at the time of initial prescription	JBI: 8/10
Thompson et al. (2021) [6]	Nationwide cohort study (Denmark)	*N* = 67,419 (Older adults ≥ 75 yrs, Primary prevention)	Discontinuation defined as no statin refill for ≥180 days	Advanced age, absence of acute cardiovascular events, polypharmacy	Statin discontinuation was associated with an increased risk of MACE (HR = 1.33; 95% Cl: 1.19–1.49) and all cause mortality (HR = 1.35; 95% Cl: 1.25–1.46)	NOS: 8/9
Giral et al. (2019) [7]	Nationwide population-based cohort (France)	*N* = 120,173 (Continuous statin users turning 75 yrs, Primary prevention)	Statin discontinuation at age 75 (no prescription refill for 3 consecutive months)	Age-related deprescribing, absence of recent cardiovascular symptoms	Discontinuation associated with a 33% increase in risk of cardiovascular admission (HR = 1.33; 95% Cl: 1.24–1.41)	NOS: 9/9
Vitturi and Gagliardi (2021) [8]	Retrospective cohort study (Brazil)	*N* = 412 (Secondary prevention—Acute ischemic stroke)	Statin withdrawal during acute stroke phase or poor pre-stroke adherence	In-hospital medication disruption, lack of standardized acute stroke protocols	In-hospital statin withdrawal was independently associated with worse functional outcome at 3 months (OR = 2.41; 95% Cl: 1.35–4.31) and higher mortality	NOS: 7/9
Jeong et al. (2020) [9]	Population-based retrospective cohort (South Korea)	*N* = 10,870 (Secondary prevention—POST-PCI patients)	Statin discontinuation within 1 year following percutaneous coronary intervention	Asymptomatic status post-PCI, fear of long-term side effects	Discontinuation markedly increased 3-year overall mortality (HR = 2.15; 95% Cl: 1.54–3.00) and recurrent MI rates	NOS: 8/9
Tong et al. (2015) [10]	Observational retrospective cohort (China)	*N* = 286 (Secondary prevention—Thrombolyzed ischemic stroke)	Statin withdrawal beyond acute phase (cessation within 30 days post-stroke)	Fear of hemorrhagic transformation, clinician preference	Statin withdrawal beyond acute phase was an independent predictor of poor 90-day functional outcome (OR = 2.68; 95% Cl: 1.31–5.47)	NOS: 7/9
Kim et al. (2015) [13]	Prospective observational cohort (South Korea)	*N* = 9330 (Secondary prevention—Acute MI)	Post-discharge statin discontinuation (cessation at follow-up)	Lack of perceived need, side effect concerns, lack of post-MI follow-up	Discontinuation was an independent predictor of 1-year MACE (HR = 1.71; 95% Cl: 1.32–2.22) and all-cause mortality (HR = 2.38; 95% Cl: 1.51–3.74)	NOS 8/9
Hovingh et al. (2016) [39]	Multi-country cross-sectional clinician survey (Europe, USA, Canada)	*N* = 1232 (Clinicians treating CVD, Primary and Secondary)	Practice-level statin discontinuation or dose reduction due to SAMS	Statin-associated muscle symptoms (SAMS), patient anxiety, muscle complaints	SAMS accounted for up to 75% of all statin discontinuations in daily clinical practice; high clinician burden in managing intolerance	JBI: 8/9
Seaman et al. (2020) [31]	Retrospective population cohort (Australia)	*N* = 10,783 (Older adults ≥ 65 yrs, Primary and Secondary)	Discontinuation following a co-payment policy increase (gap > 60 days)	Increased out-of-pocket medication costs, low GP visit frequency	Frequent GP visits were protective against cost-driven discontinuation (OR = 0.72; 95% CI: 0.61–0.85)	NOS: 8/9
Hickson et al. (2017) [32]	Retrospective cohort study (USA)	*N* = 15,317 (Older adults ≥ 65 yrs, Post-AMI setting)	Low adherence or discontinuation (PDC < 80% within 1 year post-AMI)	Absence of timely primary care or cardiologist follow-up after hospital discharge	Early post-discharge follow-up with primary care physicians or cardiologists significantly reduced the risk of statin discontinuation	NOS: 7/9
W Clark et al. (2019) [41]	Prospective interventional study (USA)	*N* = 54 (Community pharmacy setting, Primary and Secondary)	Non-adherence driven by knowledge gaps	Lack of medication education, misconceptions regarding statin safety	Pharmacist-led education significantly improved patient knowledge scores (*p* < 0.001) and adherence quality metrics	JBI: 7/9
Sessa et al. (2018) [33]	Spontaneous reporting system analysis (FAERS) (Italy/Global)	*N* = 14,821 (Adverse drug reaction reports)	Discontinuation directly triggered by adverse drug reactions (ADRs)	Muscle-related symptoms, gastrointestinal discomfort, preventable drug–drug interactions	Most ADR-related discontinuations were deemed preventable; highlights the necessity of monitoring polypharmacy and potential interactions	JBI: 7/9
Perez de Isla et al. (2024) [34]	Real-world retrospective database analysis (Italy)	*N* = 8412 (Primary and Secondary prevention)	Non-adherence (PDC < 80%) or treatment abandonment	High pill burden (free-combination vs. single-pill combination)	Single-pill combination (Rosuvastatin/Ezetimibe) significantly improved long-term adherence and reduced discontinuation compared to free combinations (HR = 0.68; 95% CI: 0.61–0.76)	NOS: 8/9
Yamamoto et al. (2023) [17]	Retrospective multicenter cohort (Japan)	*N* = 12,021 (Secondary prevention—Post-coronary revascularization)	Statin discontinuation at 1 year post-PCI/CABG	Advanced age, absence of acute coronary syndrome presentation, side effect concerns	Discontinuation was independently associated with a significantly higher risk of MACE (HR = 1.48; 95% CI: 1.21–1.81) and all-cause mortality	NOS: 8/9
Lilly et al. (2014) [25]	Retrospective cohort study (US Military Health System) (USA)	*N* = 46,249 (Primary and Secondary prevention)	Non-persistence (PDC < 80% or treatment gap > 90 days)	Perception of cognitive or psychological side effects, low baseline CVD risk	Non-persistent statin users showed higher rates of psychological and cognitive complaints, though causality was unconfirmed	NOS: 7/9
Booth et al. (2017) [30]	Retrospective cohort (Medicare beneficiaries) (USA)	*N* = 57,092 (Older adults ≥ 65 yrs, Post-MI setting)	Statin discontinuation (gap > 60 days) or non-persistence within 1 year post-MI	Absence of timely physician follow-up, low initial dose intensity, female sex, polypharmacy	Discontinuation occurred in nearly 30% of post-MI patients within 1 year; re-initiation was low and associated with higher recurrent MI risk (HR = 1.38; 95% CI: 1.22–1.56)	NOS: 8/9
Tokgözoğlu et al. (2016) [40]	Observational, cross-sectional (Turkey)	*N* = 532 (Patients with hypercholesterolemia who discontinued statin therapy)	Self-reported or physician-confirmed cessation of statin treatment	Patient-driven decisions (73.7%), higher education level, negative TV media coverage regarding side effects, lack of knowledge about hypercholesterolemia risks	Major driver of self-initiated cessation; switch to non-pharmacological/herbal remedies; no direct hard clinical endpoint HR measured (focus on patient-reported behaviors and nocebo drivers)	JBI: 6/8
Engell et al. (2023) [35]	Qualitative/Mixed-methods (Ireland/UK)	*N* = 23 PCPs and 20 Patients (Primary care physicians and patients on/discontinuing statin therapy)	Self-reported statin non-adherence, treatment interruption, or clinical deprescribing	Communication gaps regarding CV risk, fear of muscle/organ adverse effects, lack of perceived necessity in primary prevention, age/frailty considerations	Qualitative outcome: Unplanned self-discontinuation without clinician advice; reluctance to re-initiate; misalignment between patient risk perception and physician management goals	NOS: 7/9
Tanner et al. (2017) [42]	Qualitative nominal group technique (NGT) (USA)	*N* = 24 (Primary care physicians managing statin intolerance/discontinuation)	Physician-reported patient statin discontinuation and barriers to re-initiation	Patient-reported muscle/joint symptoms (47% weighted votes); patient fear and apprehension during re-challenge (70% weighted votes)	Statin re-challenge failed or hindered in 70% of cases due to patient fear; clinical management deadlock despite physician attempts to re-initiate	JBI 8/10

**Table 5:** Determinants of Statin Discontinuation and Non-Adherence.

No.	Methodology and Exposure Definition	Primary Quantitative Results	Key Identified Determinants and Findings	Citation
1.	Follow-up tracking of selective statin cessation vs. ongoing concurrent therapy	20.0% (*n* = 5819) discontinued statins exclusively	Mean age: 76.5 yrs; 60% men. Cessation driven by polypharmacy reduction and fear of side effects.	[5]
2.	Statin therapy at discharge (users vs. non-users) following an ESUS event.	Recurrent Ischemic Stroke: HR = 0.58 (95% CI: 0.38–0.88, *p* = 0.011) All-Cause Mortality: HR = 0.51 (95% CI: 0.31–0.84, *p* = 0.008) Major Adverse Cardiovascular Events (MACE): HR = 0.55 (95% CI: 0.38–0.79, *p* = 0.001)	Statin use upon discharge after ESUS is significantly associated with a reduced risk of recurrent ischemic stroke, lower overall mortality, and fewer MACE. Statin therapy acts as a strong protective determinant against adverse long-term cardiovascular outcomes in ESUS patients.	[11]
3.	Pharmacy refills within 90 days post-discharge; 90-day interval audits over 12 yrs	27.1% non-initiators at 90 days; 36.0% non-persistent over 12 yrs	Female sex and older age associated with lower therapy continuation.	[12]
4.	Discontinuation = first ≥ 90-day gap after estimated prescription end	47% discontinued (primary prevention); 41% discontinued (secondary prevention)	Younger age, female sex, and lower socioeconomic status increase discontinuation risk. (72–75% restarted later).	[16]
5.	Questionnaire assessing medication beliefs and willingness to deprescribe	95% willing to deprescribe statins; 94% concerned about side effects	85% desired stopping ≥ 1 regular drug; high side effect concern drives willingness to deprescribe.	[18]
6.	Discontinuation defined as no refill within 12 months post-first prescription	12.0% 1-year discontinuation rate	High co-payment increases cessation risk. Older age, vascular comorbidities, and higher BMI reduce risk.	[22]
7.	Adherence measured via self-reports and pharmacy refill data	40–70% doubted necessity/efficacy; 20–35% reported joint/muscle side effects	Fear/experience of muscle/joint side effects strongly tied to intentional non-adherence.	[23]
8.	Correlation between volume of negative media news stories and statin cessation rates	Significant association with negative media coverage	Younger age, female sex, and lower socioeconomic status increase discontinuation risk. (72–75% restarted later).	[24]
9.	Comparison of baseline characteristics between 2-year persistent and non-persistent users	Non-persistent at 2 years	Female sex, younger age, and fewer comorbidities correlate with non-persistence.	[25]
10.	Statin fill within 30 days post-MI discharge; tracking of low, med, high-dose cessation	15.4% total discontinuation; Discontinuation: 17.4% (low), 15.3% (med), 15.0% (high dose)	Older age, prior statin naivety and high comorbidity burden drive cessation. 53.7% restarted within 1 yr.	[30]
11.	Patient questionnaire evaluating experiences, concerns, and communication gaps	6.0% completely stopped therapy	Female sex, age < 65 yrs and low CVD burden drive cessation. Widespread patient-physician communication gaps reported.	[36]

MI—myocardial infarction.

**Table 6:** Reasons for statin non-adherence.

No.	Methodology and Exposure Definition	Primary Quantitative Results	Key Identified Determinants and Findings	Citation
1.	Audio-recorded interviews; thematic coding via ATLAS.ti 8.0	Qualitative identification of core themes for primary non-adherence	Primary desire to try alternative/lifestyle remedies first Good self-rated health and feeling “too young” for statins Distrust of physicians and belief that genetic cholesterol is untreatable	[19]
2.	Treatment Perception Questionnaire (TMI) and pharmacy refill tracking	71%: perceived limited statin efficacy 49%: doubted statin prevention of CVD 40%: felt statins were unnecessary	62% feared musculoskeletal side effects (45% experienced) 29% feared fatigue/insomnia (19% experienced) 18% feared GI side effects (10% experienced)	[23]
3.	Survey on perceived benefits and drivers of statin discontinuation	62.6%: cited financial savings as primary benefit	24.6% expected QoL improvement post-discontinuation	[37]
4.	Observational, national cross-sectional study (STAY study) in patients with hypercholesterolemia who discontinued statin therapy (*N* = 532)	73.7% of discontinuation decisions made by patients (vs. 9.0% by family physicians, 17.3% by specialists) 80.4% discontinuation rate in patients with at least secondary education vs. 69.7% in lower education (*p* = 0.022) Negative TV coverage of side effects: 38.0% hepatic, 33.8% renal, 32.9% muscular	Discontinuation is overwhelmingly patient-driven; higher educated patients are more likely to stop statins independently and switch to alternative/non-drug therapies. Negative information from TV programs regarding potential organ/muscle side effects is the leading external driver of discontinuation.	[40]
5.	Qualitative online nominal group technique (NGT) with primary care physicians (PCPs); weighted voting process	47% of weighted votes: side effects as the main reason for statin discontinuation 31% of weighted votes: statin re-challenge as the most common clinician action 70% of weighted votes: patient fear of side effects as the main barrier to re-initiation	Musculoskeletal and joint-related symptoms were identified as the single primary reason reported by patients. While PCPs frequently attempt statin re-challenge, patient reluctance remains the major obstacle.	[42]
6.	Revised Patients’ Attitudes Towards Deprescribing (rPATD) questionnaire for statins	38%: doubted stopping statins triggers CVD events 6%: believed statins are unnecessary	47% experienced/suspected side effects (22% concerned) 22% willing to stop to “test how they feel” 13% reported daily pill inconvenience	[38]

CVD—cardiovascular disease, rPATD—revised Patient Attitudes Towards Deprescribing, TMI—Tailored Medicine Inventor.

**Table 7:** Clinical and Economic Consequences of Statin Discontinuation.

No.	Methodology and Exposure Definition	Primary Quantitative Results	Key Identified Determinants and Findings	Citation
1.	Analysis of statin adherence tracking long-term survival and heart failure hospitalizations	31.7% (*n* = 9204) discontinued statin therapy during follow-up	Statin discontinuation significantly increased mortality risk (HR 1.15) and heart failure hospitalizations (HR 1.24) No significant effect seen on cerebrovascular or ischemic heart disease	[5]
2.	MACE risk assessment comparing statin continuation vs. discontinuation in elderly patients	Discontinuation rates: 30% (primary prevention) and 25% (secondary prevention)	Statin discontinuation significantly increased MACE risk in both primary (HR 1.32) and secondary prevention (HR 1.28)	[6]
3.	Inclusion required MPR ≥80% over prior 2 years and no previous history of CVD	33% relative increase in cardiovascular hospital admissions following discontinuation	Abrupt statin discontinuation in elderly primary prevention patients significantly impairs cardiovascular protection	[7]
4.	Statin adherence measured by MMAS-8 scale; tracking functional outcomes (mRS), recurrence, and mortality	Adherence levels: 21.8% no statin, 34.9% poor, 9.1% intermediate, 35.0% good	Good adherence significantly correlated with better functional recovery and lower stroke recurrence (*p* < 0.05) Statin withdrawal and poor adherence increased stroke recurrence risk	[8]
5.	Exposure classified into 3 periods: “statin period”, “statin withdrawal period”, and “no statin period”	29.0% overall primary outcome event rate (MI, revascularization, stroke, or all-cause death)	Continuous statin use significantly reduced primary outcome risk (adj HR 0.72, *p* < 0.001) Statin withdrawal nearly doubled event risk (adj HR 1.87, *p* < 0.001)	[9]
6.	Divided into 3 cohorts: reference (no statin), continued (≥3 weeks), and withdrawal (<3 weeks post-stroke)	Statin withdrawal predicted worse 3-month outcomes (*p* = 0.004) and fewer favorable outcomes (*p* = 0.002)	In cardioembolic stroke, statin withdrawal significantly reduced favorable outcomes (*p* = 0.027) and elevated mortality/poor outcome risk (*p* = 0.010)	[10]
7.	National health registries tracking hospital admissions, mortality, and prescriptions (statin fill within 90 days post-discharge)	27.1% discontinued statin therapy post-ischemic stroke 12-year all-cause mortality: 56.8% (no statin) vs. 48.6% (statin)	Discontinuation led to significantly higher MACCE risk (50.0% vs. 46.3%) and cardiovascular death (34.8% vs. 30.2%)	[12]
8.	Propensity score matching comparing statin withdrawal (*n* = 603) vs. non-withdrawal (*n* = 3204)	Withdrawal group presented with older age, lower BMI, and worse biomarker profiles	Statin withdrawal dramatically increased overall all-cause mortality (HR 3.45) and cardiac mortality (HR 4.65)	[13]

BMI—body mass index, MACCE—major adverse cardiac and cerebrovascular events, MACE—Major adverse cardiac events, MI—myocardial infarction, MMAS-8 scale—Morisky Medication Adherence Scale.

**Table 8:** Role of the physician and health care system in statin adherence.

No.	Methodology and Exposure Definition	Primary Quantitative Results	Key Identified Determinants and Findings	Citation
1.	Internet-based 88-item questionnaire evaluating drug interactions and decision-making	93% took polypharmacy, but <15% were informed about drug interactions 95% valued personalized choices, but 76% had zero input	Poor patient-physician communication and lack of shared decision-making contribute significantly to patient dissatisfaction	[36]
2.	Qualitative and quantitative assessment of patient experiences and deprescribing attitudes	41%: reluctant to discontinue statin therapy 22%: willing to discontinue statin therapy	Patient-physician trust creates reluctance to stop statins even when deprescribing is clinically considered	[38]
3.	Questionnaire assessing SAS diagnostic criteria, treatment adjustments, and incidence management	72% of suspected SAS cases had verified muscle symptoms 6% total estimated SAS prevalence 52% managed SAS via dose reduction + add-on therapy	Clinicians frequently maintain statin therapy through dose reduction and combination regimens rather than complete discontinuation	[39]
4.	Linked hospital, Medicare, and mortality data tracking 6-month post-index statin status	GP visit frequency directly predicted continuation rates over 1-year follow-up	Frequent primary care contact (≥3 visits/year vs. 1 visit) reduced statin withdrawal risk by 47%	[31]
5.	Assessment of statin adherence changes comparing 6 months pre- vs. 6 months post-AMI	64.0%: unchanged adherence 19.7%: increased adherence 16.3%: decreased adherence	Acute cardiovascular events (AMI) act as a strong trigger to improve adherence, though a substantial minority still experiences decline	[32]
6.	Pharmacist-led educational intervention on statin risk-benefit profiles	80% identified ≥1 statin benefit post-intervention Willingness to fill statin prescriptions rose from 30% to 80%	Structured pharmacist education directly addresses patient misconceptions and dramatically boosts initial filling willingness	[41]
7.	Regional registry tracking suspected statin-induced adverse drug reactions (ADRs)	81.4% of statin withdrawals were caused by non-serious ADRs with positive prognoses	Unnecessary statin cessation is predominantly driven by minor, non-serious adverse effects that could be managed clinically	[33]
8.	Qualitative/mixed-methods study assessing primary care experience, attitudes, and decision-making regarding statin continuation, deprescribing, and adherence	Qualitative synthesis of PCP and patient perspectives on statin discontinuation dynamics and primary care barriers	Misalignments between clinician intentions and patient understanding regarding cardiovascular risk reduce persistence.	[35]
9.	Adherence comparison: Single-Pill Combination (SPC) vs. Multi-Pill Combination (MPC)	Adherence rate: 75.2% (SPC) vs. 51.8% (MPC), *p* < 0.001	Simplifying treatment regimens via single-pill fixed combinations significantly improves patient adherence	[34]

ADR—adverse drug reactions, AMI—acute myocardial infarctions, GP—general practitioner, SAS—statin-associated symptoms.

## Data Availability

This study is based on data from previously published studies. All data supporting the findings of this review are available in the cited articles.

## Supplementary Materials

The following supporting information can be downloaded at: https://www.mdpi.com/article/10.3390/metabo16090603/s1, Table S1. Database search strategies, and screening limits applied in the systematic review.

## Abbreviations

AMI	acute myocardial infarction
HDL	high-density lipoprotein
HMG-CoA	3-hydroxy-3-methylglutaryl coenzyme A reductase
LDL	low-density lipoprotein
PCI	percutaneous coronary intervention
SAS	statin-associated symptoms
