# Supplementary material for: The Statin Paradox: Drivers and Consequences of Therapy Discontinuation
Source: Metabolites. 2026 Aug 24;16(9):603. doi: 10.3390/metabo16090603 (PMC13609749; doi:10.3390/metabo16090603)
Supplement: Supplementary file 1 [file metabolites-16-00603-s001.zip › metabolites-4446944-supplementary.pdf]

The Statin Paradox: Drivers and Consequences of Therapy Discontinuation

Adrianna Dylik, Mikołaj Musiał, Michał Radke, Dominika Tuzimek, Dominika Rogoża, Bartosz Czekała, Anna Wawrzyniak, Nadzeya Zhuk, Katarzyna Skrypnik, Damian Skrypnik

Supplementary Table S1. Database search strategies, and screening limits applied in the systematic review.

Database	Detailed Search Strategy	Applied Filters & Limits	Number of results
PubMed (MEDLINE)	("statins" AND ("Medication Adherence" OR "discontinuation" OR "non-adherence" OR "nonadherence" OR "deprescribing") AND ("Cardiovascular Diseases" OR "Mortality" OR "cardiovascular events" OR "myocardial infarction" OR "stroke" OR "mortality"))	Original research articles only; Humans only (animal studies excluded); English language; Publication date: 01.01.2014–15.07.2024	1057
Google Scholar	statins AND (discontinuation OR non-adherence OR deprescribing) AND (cardiovascular OR mortality)	Original research articles only; Humans only (animal studies excluded); English language; Publication date: 01.01.2014–15.07.2024	504
Web of Science	statins AND (discontinuation OR non-adherence OR deprescribing) AND (cardiovascular OR mortality)	Original research articles only; Humans only (animal studies excluded); English language; Publication date: 01.01.2014–15.07.2024	471
